# CRYSPER and CUREIPES: two new molecular tools enabling analysis of RNA splicing and C-to-U RNA editing-inducible protein expression at single cell resolution

**DOI:** 10.1093/nar/gkag899

**Published:** 2026-09-24

**Authors:** Magnus Harnau, Yulia Novykova, Jacqueline Weicht, Barbara Schweissthal, Leonie Emde, Julia Brach, Josephine Haake, Laura Steenpass, Steffen Fricke, Jochen C Meier

**Affiliations:** Division Cell Physiology, Institute for Cell- and Neurobiology, Technical University Braunschweig, Spielmannstr. 7, D-38106 Braunschweig, Germany; Division Cell Physiology, Institute for Cell- and Neurobiology, Technical University Braunschweig, Spielmannstr. 7, D-38106 Braunschweig, Germany; Division Cell Physiology, Institute for Cell- and Neurobiology, Technical University Braunschweig, Spielmannstr. 7, D-38106 Braunschweig, Germany; Division Cell Physiology, Institute for Cell- and Neurobiology, Technical University Braunschweig, Spielmannstr. 7, D-38106 Braunschweig, Germany; Division Cell Physiology, Institute for Cell- and Neurobiology, Technical University Braunschweig, Spielmannstr. 7, D-38106 Braunschweig, Germany; Division Cell Physiology, Institute for Cell- and Neurobiology, Technical University Braunschweig, Spielmannstr. 7, D-38106 Braunschweig, Germany; Department Human and Animal Cell Lines, Leibniz Institute DSMZ–German Collection of Microorganisms and Cell Cultures GmbH, Inhoffenstr. 7b, 38124 Braunschweig, Germany; Department Human and Animal Cell Lines, Leibniz Institute DSMZ–German Collection of Microorganisms and Cell Cultures GmbH, Inhoffenstr. 7b, 38124 Braunschweig, Germany; Division Cell Physiology, Institute for Cell- and Neurobiology, Technical University Braunschweig, Spielmannstr. 7, D-38106 Braunschweig, Germany; Division Cell Physiology, Institute for Cell- and Neurobiology, Technical University Braunschweig, Spielmannstr. 7, D-38106 Braunschweig, Germany

## Abstract

RNA splicing and editing contribute to functional diversification of proteins encoded by single genes. Previous and current research primarily focus on sequencing of bulk material and bioinformatics to identify editing and splice sites, but these technologies ignore the role of individual cell types in the regulation of RNA splicing and editing. We present two new molecular tools for analysis of canonical and noncanonical RNA splicing (CRYSPER) and human Apobec1-dependent C-to-U RNA editing-inducible protein expression (CUREIPES). They use ratiometric fluorescence analysis for single-cell readout of RNA processing. Using CRYSPER, the results show noncanonical splice site usage in different cell types and demonstrate that the second intronic position can be a wobble base. Cellular heterogeneity of cryptic splice site utilization indicates cell type-specific control of this type of RNA processing and reveal a novel dimension of the regulation of RNA splicing. Thus, CRYSPER can be the new tool for research on canonical and noncanonical RNA splicing. Based on CRYSPER, CUREIPES was developed and is the first molecular tool that enables human Apobec1-dependent C-to-U RNA editing-inducible protein expression, which can be used to counteract pathological alteration of C-to-U RNA editing through cell-autonomous expression of editing-regulatory proteins, or of any other protein of interest.

## Graphical Abstract

**Graphical Abstract:**
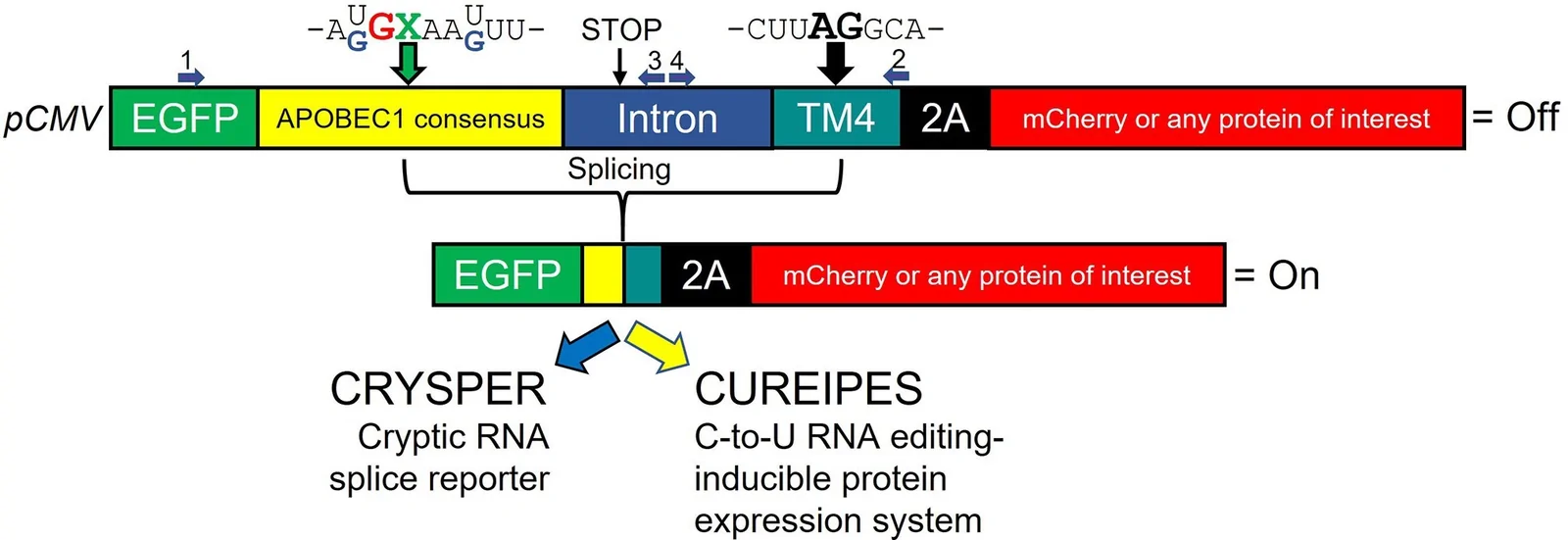

## Introduction

**Figure 1.**
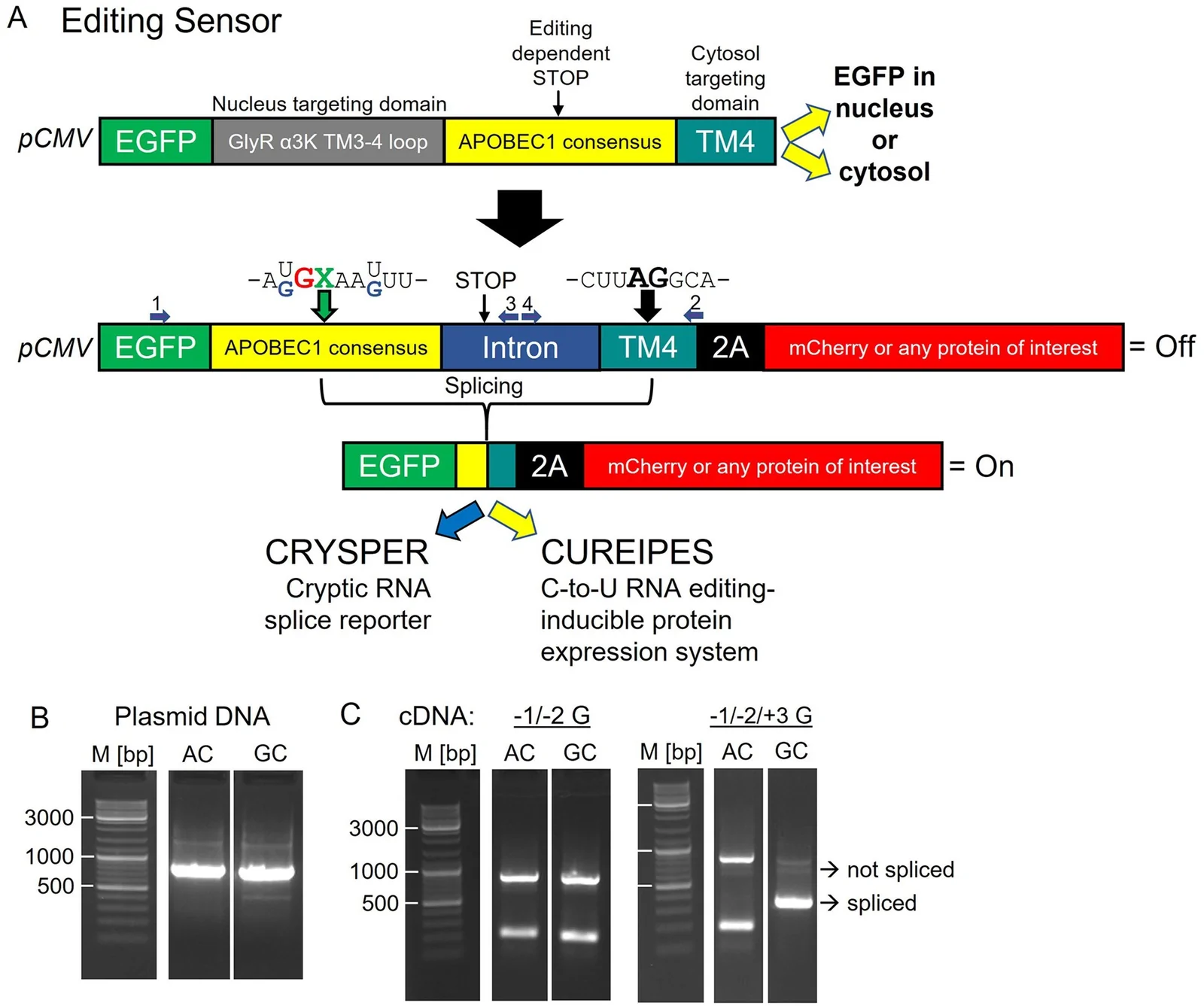
Splice and editing reporter development, mechanism and verification of the anticipated splicing in the sensor constructs. (**A**) Scheme depicting the published EdSensor [7], the modified, newly developed cryptic splice reporter (CRYSPER) and the C-to-U RNA editing inducible protein expression system (CUREIPES). The working principle of the EdSensor (above) is based on the translocation of EGFP into the nucleus upon Apobec1/ACF-dependent C-to-U RNA editing. Editing would generate a stop codon, truncating the dominant cytosol-directing domain and leaving the NLS-containing domain. Therefore, the fluorescence ratio between the cell nucleus and the cytoplasm reveals the relative editing amount. Reporter modifications started by mutating the Apobec1 consensus sequence at three positions (red and blue) to resemble a 5´-splice donor which is followed downstream by a synthetic intron (Supplementary Fig. S1) derived from *Glrb* and *Gphn* introns [31, 32]. If splicing occurs, the intronic STOP codon is removed, creating an open reading frame with 2A_mCherry located downstream of the TM4 domain of the GlyR α3k loop. EGFP is consecutively expressed and can be utilized for normalization. Consequently, the mCherry/EGFP ratio represents the relative amount of splicing in the construct´s mRNA. However, editing was absent and noncanonical splicing was observed (CRYSPER), while reverting two of the mutations (blue) resulted in editing induced splicing (CUREIPES). Arrows depict the primers that were utilized to investigate the splicing pattern (1 [GGCATCGACTTCAAGGAGG], 2 [CGACCGGTGGATCCAGAGC] → Fig. 3A/C) and isolate the intron (3 [ATCTGATGGTGATTATCTGATACA], 4 [GATCAAGAGTGAAACTGTTCCAT] → Fig. 3B/D). (**B**) Gel electrophoresis image of PCR-amplified plasmid DNA used as a size control for the 837 bp long unspliced band. (**C**) PCR amplification of complementary DNA (cDNA) isolated from reporter-expressing HEK293T cells. On the left, the −1/−2G mutated and on the right the triple mutated sensor was utilized, each with the negative control AC and the editable GC (C→U) splice donor. Splicing with the expected donor in the Apobec1 consensus sequence yields a 368 bp long band, while utilization of an unexpected donor in the GlyR α3K TM3–4 loop resulted in the 189 bp band.

RNA editing diversifies genome-coded protein functions by enzymatic deamination of cytidine or adenine nucleobases in messenger RNA (mRNA). In recent years, it turned out that alterations in RNA editing may play a key role in a plethora of idiopathic human diseases (for overview see [1, 2]). In particular, recent experimental evidence suggests that increased mRNA editing in the hippocampus is involved in epilepsy [3, 4]. However, experiments also demonstrated that functional consequences of changes in RNA editing have to be investigated at the single cell level because cell type-specific expression of a C-to-U RNA edited target in a transgenic mouse model of epilepsy revealed striking differences in the symptomatology of the mice [5, 6]. Thus, molecular tools for detection of RNA editing at the single cell level are required. Some tools for monitoring C-to-U RNA editing in single cells have already been presented [7–9]. The EdSensor reveals the relative Apobec1/ACF-dependent C-to-U RNA editing amount by the ratio of GFP in the cell nucleus to the cytoplasm. It functions by directing the fluorescent protein to the cytosol via a dominant cytosol-directing domain. When editing induces a stop codon, translation of this downstream domain is prevented, leaving the nuclear localization sequence- (NLS)-containing domain to drive nuclear translocation (Fig. 1A) [7]. However, no system was yet established that induces protein expression upon editing [10]. The mechanism of the editing event involves the melting of the secondary stem-loop structure of the *ApoB* mRNA upon ACF binding and thereby providing access to the recruited Apobec1 deaminase catalyzing the hydrolytic reaction [11].

Another important mRNA maturation step in eukaryotes is splicing, which involves the removal of noncoding introns to join the coding exons. This process also allows for protein diversification by producing multiple transcripts from a single gene via exon skipping, intron retention or selection of different splice sites. Therefore, alternative splicing can generate isoforms with altered features to extend the proteome [12]. The reaction is catalyzed by the spliceosome, a dynamic molecular machine consisting of several uridine-rich small nuclear ribonucleoprotein particles (snRNPs). After their recognition of the core intronic signals, such as the 5´-donor, 3´-acceptor splice sites, and the branch point adenosine, it leads to two consecutive transesterification reactions, resulting in an intron lariat and the spliced mRNA [13, 14]. Although the most 5´-donor splice sites encompass the dinucleotide GU at the first intronic positions, the noncanonical donor GC constitutes ∼0.5% of mammalian splice sites [15, 16]. Noncanonical splice site usage is part of the umbrella term cryptic splicing, which covers all kinds of aberrant splicing [17]. Although, cryptic splice sites are normally referred to when mutations activate nearby pseudo splice sites, in this study deviations from the consensus motifs, especially the aberrant dinucleotides, are implied. For instance, a conserved GA donor was described in the *FGFR* genes [18] and some organisms show an increased amount of noncanonical GA and GG donor sites [19]. However, their mechanism remains unclear, as early mutagenesis studies demonstrated that conversion of canonical GU donor sites to noncanonical GA or GG permits the first but stalls the second catalytic step, resulting in the accumulation of lariat intermediates [20, 21]. Nevertheless, a donor hierarchy of GT > GC > GA > GG was proposed [22] and could be explained by bulging out or shifting of nucleotides during the recognition by the U1 snRNP [16, 23].

In recent years, research on cryptic splicing in regard to activation of pseudo splice sites led to the discovery of repressors such as TDP-43 in the brain [24] or RBMXL2 in the testis [25], together the most splicing-active tissues [26]. Their loss results in the formation of cryptic exons, driving neurodegeneration [27] or defects in spermatogenesis [28]. Furthermore, splicing defects in general underlie about 10%–25% of hereditary diseases [29, 30]. Therefore, studying splicing, aberrant splice site choices and regulatory elements is of special importance for a comprehensive view of cell physiology.

We present a novel molecular tool for splicing analysis in single cells with fluorescence readout and here particularly in regard to the utilization of noncanonical splice donor sites. The plasmid-encoded reporter consists of two fluorescent proteins separated by an intron containing a stop codon. Therefore, the ratio of both correlates with the relative amount of splicing in the construct. First experiments demonstrated that noncanonical splice site utilization is possible and might be not that rare than previously anticipated. Furthermore, the second intronic position should be seen as wobble base because other nucleotides are tolerated to some extent. In HEK293T cells and primary mouse hippocampal cultures (MHC), cryptic splicing was dependent on intronic regulatory elements, while canonical splicing was induced by the strong donor alone. Splicing differed in individual cells and between cell types and hence maybe due to differential regulation, toleration or control of cryptic splicing. Furthermore, we constructed a second molecular tool which initiates protein expression upon C-to-U RNA editing induced splicing of the reporter mRNA. Both tools can be used for research on splicing and editing without relying on molecular techniques, rather utilizing the advantage of fluorescence readout.

## Materials and methods

### Construct production

The Apobec1-specific RNA editing sensor construct is published [7]. Based on this plasmid, the generation of the splicing dependent protein expression system was established (Fig. 1A). For this purpose, a synthetic construct was synthesized by Biomatik (Cambridge, Ontario, N3H 4R7, Canada) and delivered subcloned in a pBluescript vector. The construct contains a core sequence with a synthetic intron directly downstream of the Apobec1 consensus [7]. The intronic core sequence (Supplementary Fig. S1) is composed of a short intronic GlyR β intron [31] and a modified gephyrin intronic sequence [32]. The Apobec1 consensus sequence was mutated at three positions −1A→G, −2T→G and +3T→G (relative to the C-to-U RNA edited site) to gain a strong splice donor site if guanines are present, as predicted by the maximum entropy model (Table 1) [33]. A plasmid with an adenine instead of guanine at position −1 served as negative control (AC). To enable protein expression (here mCherry), a cassette coding for a 2A peptide [34] and an mCherry-coding open reading frame was inserted downstream using standard restriction enzyme dependent molecular cloning. Furthermore, the GlyR α3 TM3–4 intracellular loop was deleted (Fig. 1A).

**Table 1.** Prediction of splice sites according to maximum entropy model [33]

Splice sites	Mutations	Model score
Splice donor 1 (tested as GT)	−1G	+1.96
	−1/−2G	+8.88
	−1/+3G	+9.11
	−1/−2/+3G	+11.08
Splice donor 2 (in α3 loop)	/	+8.41
Splice acceptor 1 (predicted)	/	+11.50
Splice acceptor 2 (utilized)	/	+4.62

The exchanges of the synthetic intron to the PFN1 intron2 or the GPHN exon 9 were carried out by PCR of the fragments with overlapping primers and ligation with NEBuilder cloning. The genomic template DNA was previously isolated from a HEK293T cell pellet with DirectPCR lysis reagent (Viagen, #102-T) and the manufacturer´s directions. Further alterations in the splice donor were introduced by PCRs with primers carrying the corresponding mutations and subsequent DpnI (NEB, #R0176) digestion (1 h, 37°C) before ligation.

The construct transcription is driven by the CMV promotor and bacterial selection is given by a kanamycin resistance gene.

### Molecular cloning techniques

To enable the construct production, molecular cloning procedures were performed in the following manner. PCRs for cloning were carried out with Q5 high fidelity polymerase (NEB, #M0491) and the manufacturer’s protocol. Following gel electrophoresis, the correct bands were cut out and extracted with the Monarch DNA Gel Extraction Kit (NEB, #T1120). Ligation was achieved with the NEBuilder Hifi DNA Assembly Mix (#M5520A) for 15–60 min at 50°C. Transformation in chemically ultra-competent *Escherichia coli* XL1blue by heat shock and subsequent plasmid isolation with Monarch Plasmid Miniprep (NEB, #T1110) or PureLink HiPure Plasmid Midiprep (Invitrogen, #K210005) kits yielded the described reporter constructs. Sequencing and primer production was performed by Eurofins Genomics.

### RNA

For assessment of the splicing patterns, total RNA was isolated from HEK293T cells using TRIzol reagent (Life Technologies, #15 596 018). To eliminate residual plasmid DNA, the sample was treated with RNase-free DNase (Roche, #04 716 728 001) as described previously [35]. Briefly, 50 units were incubated (30 min, 37°C) with 50 μg RNA sample and purified using RNeasy Protect Mini Kit (Qiagen, #74 124) according to manufacturer’s instructions. Complementary DNA was obtained by reverse transcription (60 min, 42°C) of 2 μg RNA with SuperScript™ II reverse transcriptase (Invitrogen, #18 064 014) and an equimolar mixture of 3′-anchored poly-T oligonucleotides (T18V, T15V, T13V). For the isolation of the synthetic intron to allow sequencing of the splice sites, DNA-digested RNA was treated for 90 min with T4 RNA Ligase 1 (NEB, #M0204) at room temperature with 1 U/µl murine RNase inhibitor (NEB, #M0314) and the manufacturer’s RNA circularization protocol. The following cDNA synthesis was completed with an intron-specific primer and the amplification was achieved using primers binding the middle of the intron in opposite directions, with 5 % DMF added. Splicing patterns were analyzed by PCR with 200 ng cDNA and subsequent sequencing of amplified regions of interest. These reactions and the PCR amplification of GAPDH and nontranscribed plasmid regions, to verify double-stranded DNA-free cDNAs, were performed with Taq polymerase (NEB, #M0267) and the manufacturer´s protocol.

### Cell culture and primary mouse hippocampal neuron isolation

HEK293T cells (DSMZ, ACC 635) were cultured in Dulbecco’s modified Eagle’s medium (DMEM) media (Gibco, #41965-039) with 10% fetal bovine serum (FBS) (Gibco, #10 500 064) and 1% pen/strep (Gibco, #15 140 122) in a humidified incubator at 37°C and 5% CO_2_. They were passaged twice a week before reaching confluency using trypsin/ethylenediaminetetraacetic acid (Sigma–Aldrich, #T2601). Cell count was determined with a Fuchs–Rosenthal chamber and cells were seeded either on tissue culture (TC) dishes for RNA isolation or on lysine-coated coverslips for fluorescence experiments. The latter was achieved by seeding 10 000 cells in 150 µl media directly onto the coverslip in 24-well plates and adding 225 µl media following their attachment after 2–5 h.

The 13 mm coverslips (VWR, #631-1578) were previously coated with 0.5 mg/ml poly-L-lysine hydrobromide (Sigma–Aldrich, #P2636) for 3 h at 37°C after treating with 10 M NaOH for 3 h at 100°C and sterilization at 180°C for 8 h.

Primary MHC were prepared from C57BL/6 mice at embryonic day 17.5 and plated on the lysine-coated coverslips at a density of 5 × 10^4^ cells per well. The MHC were incubated in Neurobasal (NB) media (Invitrogen, #21103-049) supplemented with 1× N2 solution, 1× B27 (Gibco, #17504-044), 0.5 mM L-glutamine (Gibco, #25030-024), 1% FBS, and 0.05% pen/strep (NB^+^) in the humidified incubator at 37°C and 5% CO_2_.

### Transfection

Construct expression in HEK293T cells was established by transfection with FuGENE HD transfection reagent (Promega, #E2311). Per 1.5 ml media in the TC dish or well (375 µl on coverslip), the transfection mixes contained 100 µl DMEM, 3 µl FuGENE, and 1 µg plasmid DNA.

MHC were transfected using lipofectamine 2000 (Invitrogen, #11 668 019) on DIV7. Per well, 2 µl lipofectamine and 0.8 µg plasmid DNA were separately solved in 50 µl NB media, mixed and incubated for 20 min at room temperature (RT). Neuron-containing coverslips were transferred in 300 µl prewarmed NB media without supplements and the transfection mix was added for 45 min. Afterwards, the MHC were repositioned into their old NB^+^-filled well.

### Immunochemistry and microscopy

Following two (HEK293T) or three days expression (MHC), cells were fixed using 4% paraformaldehyde (Sigma–Aldrich, #P6148)/sucrose (Carl Roth, #9097.1) for 15 min at RT and mounted using Vectashield with 4′,6-diamidino-2-phenylindole (DAPI) (Vector Labs, #H-1200). Primary hippocampal cultures were further processed for immunochemistry using antibodies to differentiate neurons and glia (anti-MAP2 1:300, Synaptic Systems, #188 004; anti-guinea pig IgG-Cy5 1:200, Dianova, #706-175-148). Both antibodies were incubated subsequently for each 45 min after permeabilization with 0.12% Triton X100 (Sigma–Aldrich, #X100) for 4 min and intermediate washing steps with 0.1% phosphate buffered saline–gelatin.

Fluorescence microscopy was performed using the laser scanning confocal microscope Leica Stellaris 5 and provided LasX software. The excitation wavelengths were 649 nm (Cy5), 584 nm (mCherry), 489 nm (eGFP), 405 nm (DAPI), and emissions were measured in the intervals of 658–720 nm (Cy5), 590–630 nm (mCherry), 494–550 nm (eGFP), or 420–460 nm (DAPI), respectively.

### Flow cytometry

Transfected HEK293T cells in 96-well plates were loaded into the Beckmann Coulter CytoflexS flow cytometer and recorded at a medium flow rate measuring 10 000 events. Nontreated cells served to set the gating parameters, and GFP- or mCherry-expressing cells were used to determine the fluorescence thresholds of the FITC and APC channels. Data acquisition and analysis was performed with CytExpert software.

### Data analysis and statistics

ImageJ [36] was utilized for the fluorescence analysis, Microsoft Excel for data collection and Graph Pad Prism 9.1 for statistical analysis. SnapGene was used to organize plasmid maps and align DNA sequences. To increase image resolution, the resulting sequence images were subsequently enhanced using AI-based upscaling, while RNA-edited transcripts were analyzed with the BEAT program [37].

Line scan fluorescence intensity was separated into cytosolic and nuclear parts of the cell according to the DAPI signal by a custom written python script. The fluorescence in the splicing constructs was measured only in the nucleus and MAP2 staining was used to distinguish neurons and glia in the primary MHC.

Data normality was assessed using the Kolmogorov–Smirnov test. Normally distributed data were analyzed using one-way analysis of variance (ANOVA) followed by Tukey’s multiple comparison test, whereas non-normally distributed data were analyzed using the Kruskal–Wallis test followed by Dunn’s multiple comparison test. For the detection of splicing outliers in the CRYSPER-Exon results, the ROUT tool [38] with 1% false discovery rate was employed.

Data are presented as mean ± standard error of the mean (SEM) with the *P*-values described as *P ≤.05; **P ≤.01;***P ≤.001; ^****^P ≤.0001.

## Results

### Construction and verification of cryptic splicing in the reporter construct

We modified the recently described Apobec1 editing sensor domain [7] at different positions relative to the edited cytidine (−1 A→G and −2/+3 T→G). Compared to the previously published editing sensor (Edsensor, 7; Fig. 1A) the Apobec1 consensus sequence was thus differentially mutated at three positions to generate a strong splice donor if Apobec1 catalyzed editing occurs. Mutation at position −1 is obligatory if C-to-U RNA editing is supposed to produce a splice donor site (GU). Mutations T→G at sites −2 and +3 were predicted (Table 1) using maximum entropy model [33]. A synthetic intron was derived from the glycine receptor β (*Glrb*) and gephyrin (*Gphn*) genes (Supplementary Fig. S1), both of which undergo alternative splicing and contribute to inhibitory neurotransmission [31, 32]. The insertion of this intron with an internal STOP codon after the mutated Apobec1 consensus sequence followed by an mCherry coding open reading frame (ORF) should yield a C-to-U RNA editing and splicing induced protein expression system (CUREIPES). Here, EGFP is expressed as transfection marker and for normalization. Editing would lead to a splicing event which removes the intronic STOP codon and initiates the mCherry expression separately by utilizing a 2A peptide (Fig. 1A).

**Figure 2.**
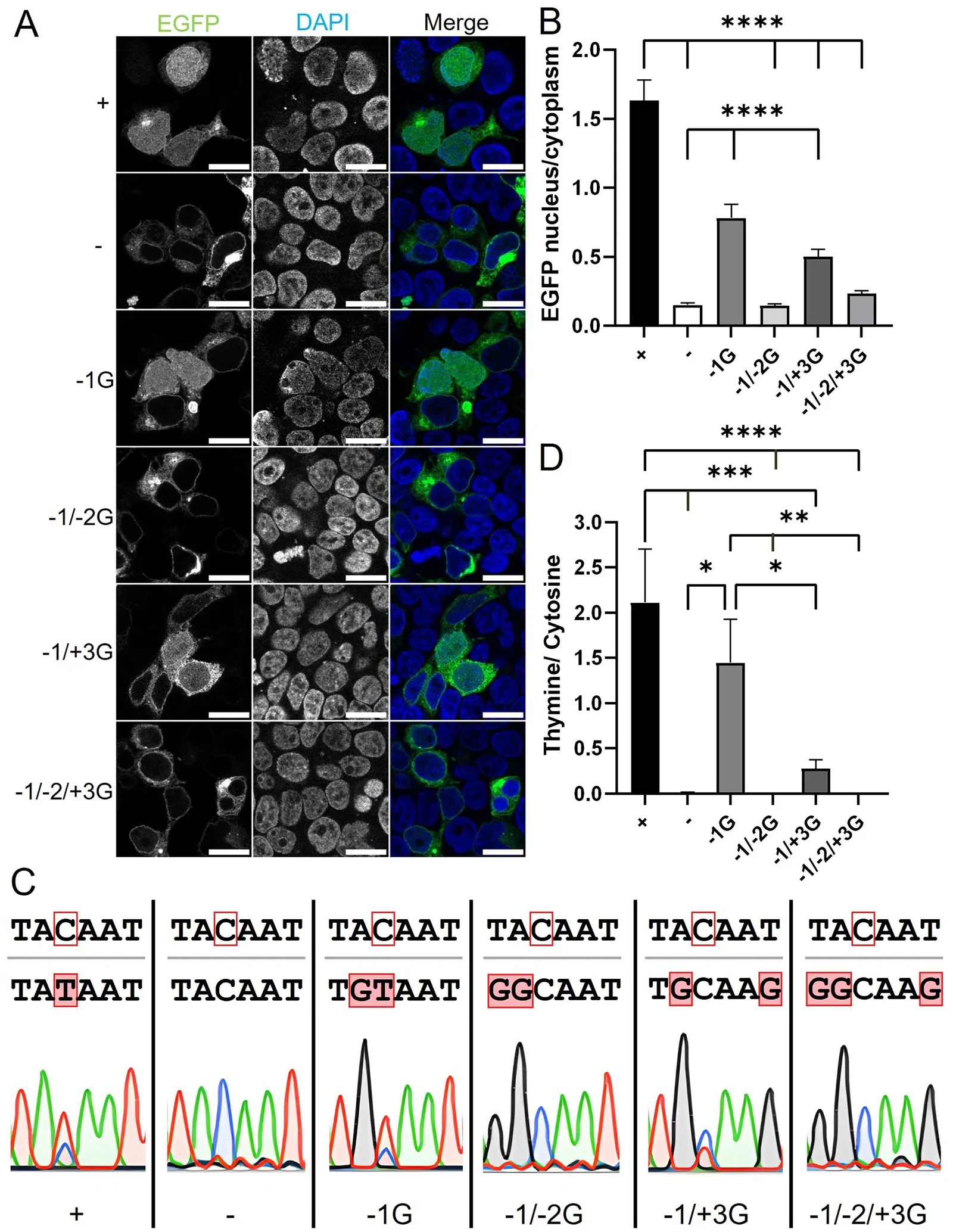
C-to-U RNA editing of the published editing sensor with the additional mutations in the Apobec1 consensus sequence. (**A**) Representative images of HEK293T cells overexpressing Apobec1 and ACF (except the negative control “−“) in addition to the EdSensor [7] with the corresponding mutations. The numbering refers to the mutated Apobec1 consensus sequence from *ApoB* in regard to the editable cytosine (position 0). The scale bars represent 20 µm. (**B**) Quantification of the ratio of EGFP fluorescence in the HEK293T cell nucleus to their cytoplasm, which depicts relative C-to-U RNA editing. EGFP is localized to the cytoplasm, but if editing occurs a stop codon is introduced, the cytoplasm-directing domain is removed and the NLS-containing domain transfers the fluorescence protein to the nucleus. The sample size was over 50 cells for each condition (*N* = 3), analysis was performed via line scan and the data are presented as mean ± SEM. (**C**) Sanger sequencing results of the isolated EdSensor-cDNA from transfected HEK293T cells. Above, the original EdSensor sequence is presented with the editable cytosine circled in red. Underneath is the obtained sequence with deviations marked in red and the bottom depicts the corresponding chromatogram in which cytosine is indicated in blue and thymine in red. C-to-U RNA editing is noticeable as the overlapping C-to-T discrepancy. (**D**) Mean ratio of T-to-C in the sequencing results of more than four independent experiments, with data extracted by the BEAT program [37] and presented as mean ± SEM. *P ≤.05; **P ≤.01; ***P ≤.001; ^****^P ≤.0001.

Splicing was verified by RNA isolation and gel electrophoresis following cDNA amplification by PCR. This revealed the 368 bp long predicted spliced band only in the triple mutated sensor (Fig. 1B). Subsequently, the GlyR α3 TM3–4 intracellular loop was deleted because it contained an additional utilized splice donor (Fig. 1B, 189 bp band, AUGAG**GU**GAGGGAG) [39]. However, fluorescence imaging (Fig. 2A/B) as well as sequencing of the mutated Edsensor cDNAs (Fig. 2C/D) after co-expression with Apobec1 and ACF in HEK293T cells revealed decreased C-to-U RNA editing. While the −1G mutation did not change the C-to-U RNA editing efficiency by Apobec1, the additional +3G mutation decreased it significantly and the −2G mutation abolished it completely. Nevertheless, splicing still occurred (Fig. 1B) and in order to analyze its pattern, an additional RNA ligase circularization step was performed after RNA isolation, resulting in the possibility to sequence the excised intron border.

**Figure 3.**
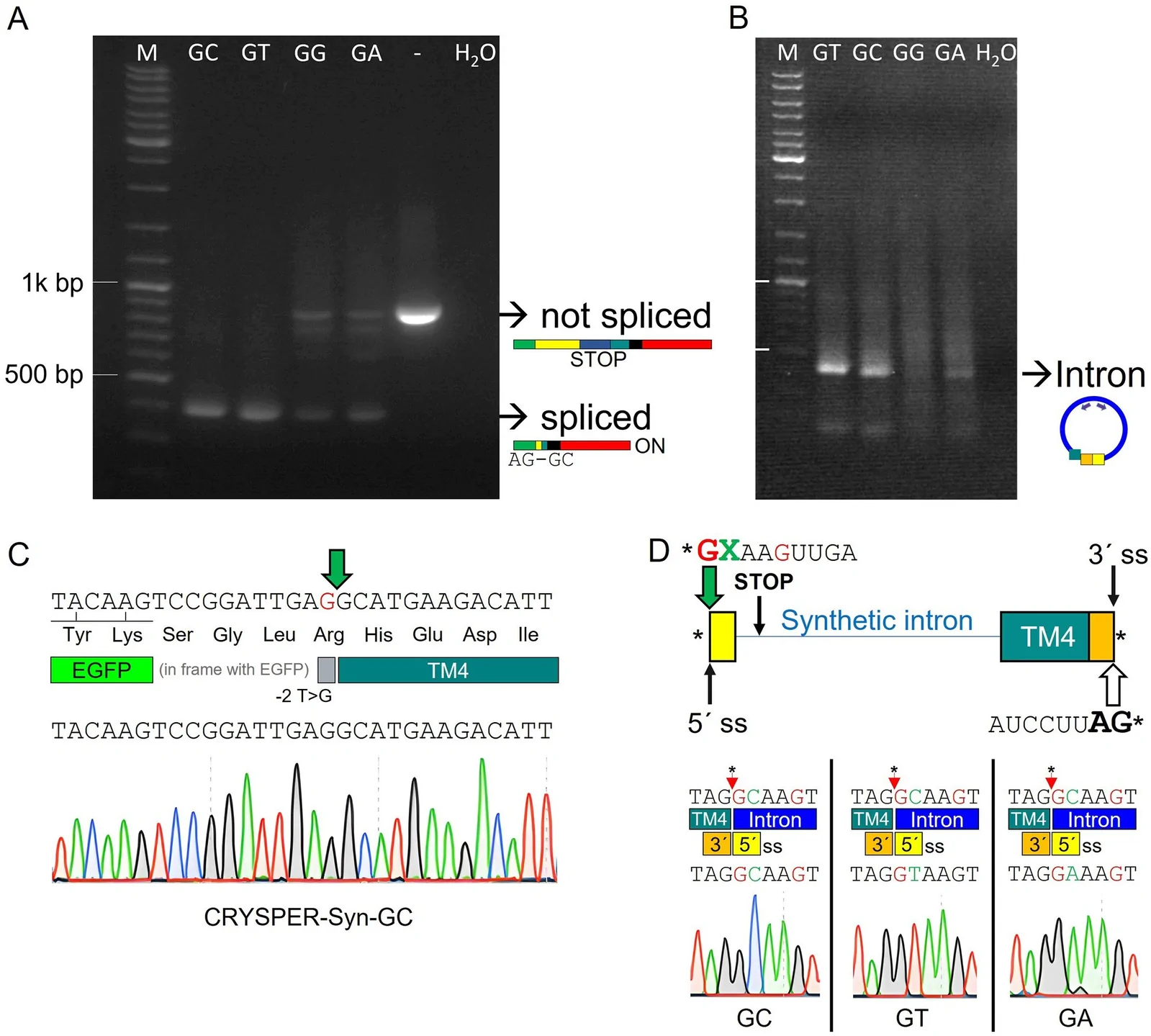
Discovery of noncanonical splice site utilization in HEK293T cells. (**A**) Gel electrophoresis image of PCR products obtained from cDNA of CRYSPER-transfected HEK293T cells with primers (refer to Fig. 1A) flanking the intron [1, 2]. Two distinct bands were revealed, the unspliced band at 866 bp which can be compared to the plasmid control (−) and the shorter 397 bp fragment generated by splicing. The DNA ladder marker (**M**) is displayed on the left and adjacent are the different CRYSPER constructs, that differ by only one base pair in the splice donor as referenced above. (**B**) Gel electrophoresis image of the isolated 469 bp long synthetic introns from reporter-expressing HEK293T cells. Initially, the isolated RNA was circularized using RNA ligase. Subsequently, the cDNA was synthesized utilizing an intron specific reverse primer [3]. PCR was then performed with primers oriented in opposite directions from the center of the intron [3, 4]. This method allowed for the inclusion of both the intronic splice donor and acceptor in the amplified DNA, enabling subsequent sequencing. (**C**) Sequencing result of the 397 bp long GC splice band extracted from the gel above (**A**). The sequence excerpt demonstrates that the intron was removed and the predicted ORF with EGFP_2A_mCherry was generated. (**D**) Sequencing results of the 469 bp long GC, GT, and GA intron bands extracted from the gel above (**B**). The scheme on top shows the components and splice sites of the synthetic intron. The displayed sequence excerpts present the splice sites of the different isolated cryptic spliced synthetic introns. The asterisks and red arrows indicate the connection made by the ssRNA ligase.

**Figure 4.**
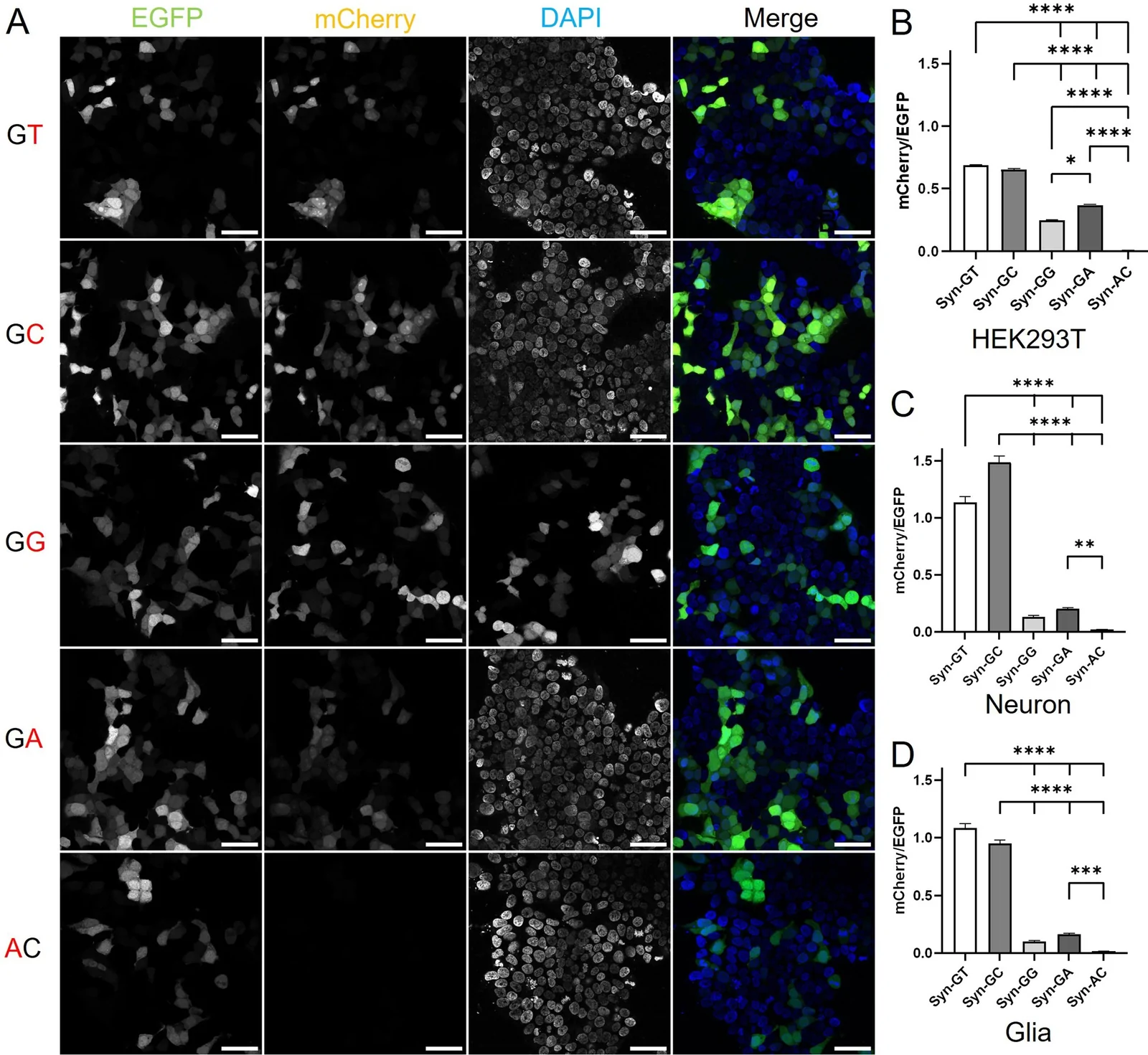
Fluorescence readout analysis of the cryptic splice reporter in HEK293T cells, MHC DIV10 neurons, and glia cells. (**A**) Representative images of HEK293T cells expressing the cryptic splice reporter with the synthetic intron and the different splice donor sites. The scale bars represent 50 µm. (**B**) Quantification (*N* = 3) of the mCherry to EGFP fluorescence ratio in the nucleus of transfected HEK293T cells, indicative for the relative splicing activity in the reporter constructs with GT (*n* = 408), GC (*n* = 563), GG (*n* = 387), GA (*n* = 397), and AC donor (*n* = 366). (**C**) Quantification (*N* = 3) of the relative splicing activity in the reporter constructs with GT (*n* = 83), GC (*n* = 75), GG (*n* = 60), GA (*n* = 64), and AC donor (*n* = 73) in transfected MHC DIV 10 neurons. Representative images are provided in Supplementary Fig. S2A. (**D**) Quantification (*N* = 3) of the relative splicing activity in the reporter constructs with (*n* = 92), GC (*n* = 73), GG (*n* = 84), GA (*n* = 100), and AC donor (*n* = 89) in transfected MHC DIV 10 glia cells. Representative images are provided in Supplementary Fig. S2B. Data are presented as mean ± SEM. *P ≤.05; **P ≤.01; ***P ≤.001; ^****^P ≤.0001.

This method exposed the splicing with the noncanonical/cryptic GC donor instead of a C-to-U RNA editing induced splicing mechanism (Fig. 3). Next, the editable C was mutated to include a GT (GU in RNA) as in canonical 5´-splice donors as well as GG and GA as more possible cryptic donors. Expression in HEK293T cells, sequencing of the resulting cDNA (Fig. 3A/C), intron isolation (Fig. 3B/D) and fluorescence analysis (Fig. 4A/B) showed that the cryptic GC splice donor was utilized by the splicing machinery similar to the canonical GT donor. Moreover, the GG and GA donor constructs were also spliced, with GA being more prominently utilized compared to the GG splice site (Figs 3 and 4A/B). However, intron isolation verified splicing only with the canonical GT and the noncanonical GC and GA donor (Fig. 3B/D).

Thus, inferentially driven by splice donor site predictions, in faith of building a C-to-U RNA editing inducible protein expression system, a cryptic splice sensor (CRYSPER) was constructed. It follows the mentioned fluorescence-based mechanism, with EGFP as transfection marker and normalization tool, while mCherry is only expressed if splicing occurs and removes the intronic STOP codon (Fig. 1A). Sequencing of CRYSPER-transfected HEK293T cells cDNA also revealed that the used splice acceptor was in a part of the transmembrane domain (TM4) of GlyR α3 (AUCCUU**AG**GCAUGAA; Fig. 3C) instead of being in the predicted region of the construct (UCUUUC**AG**GGCCUGC; Table 1).

For further verification, the experiments were reproduced in primary MHC and MAP2 staining was used to distinguish neurons (Fig. 4C and Supplementary Fig. S2A) from glia cells (Fig. 4D and Supplementary Fig. S2B). Both cell types showed comparable CRYSPER splicing with noncanonical splice site utilization and the pattern resembled that observed in HEK293T cells, although a direct comparison is limited due to differences in expression duration. Here, neurons showed higher levels of relative GC construct splicing than glia. Notably, the induced fluorescence in the GG construct was not significantly higher than the negative control AC construct (Fig. 4 and Supplementary Fig. S2).

To address whether all these effects were due to the synthetic nature of the intron, we used a natural intron as well as an exon in the following experiments.

### Insertion of a natural intron and an exon into the reporter revealed that noncanonical splice site utilization depends on intronic regulatory splicing elements rather than the splice donor itself

**Figure 5.**
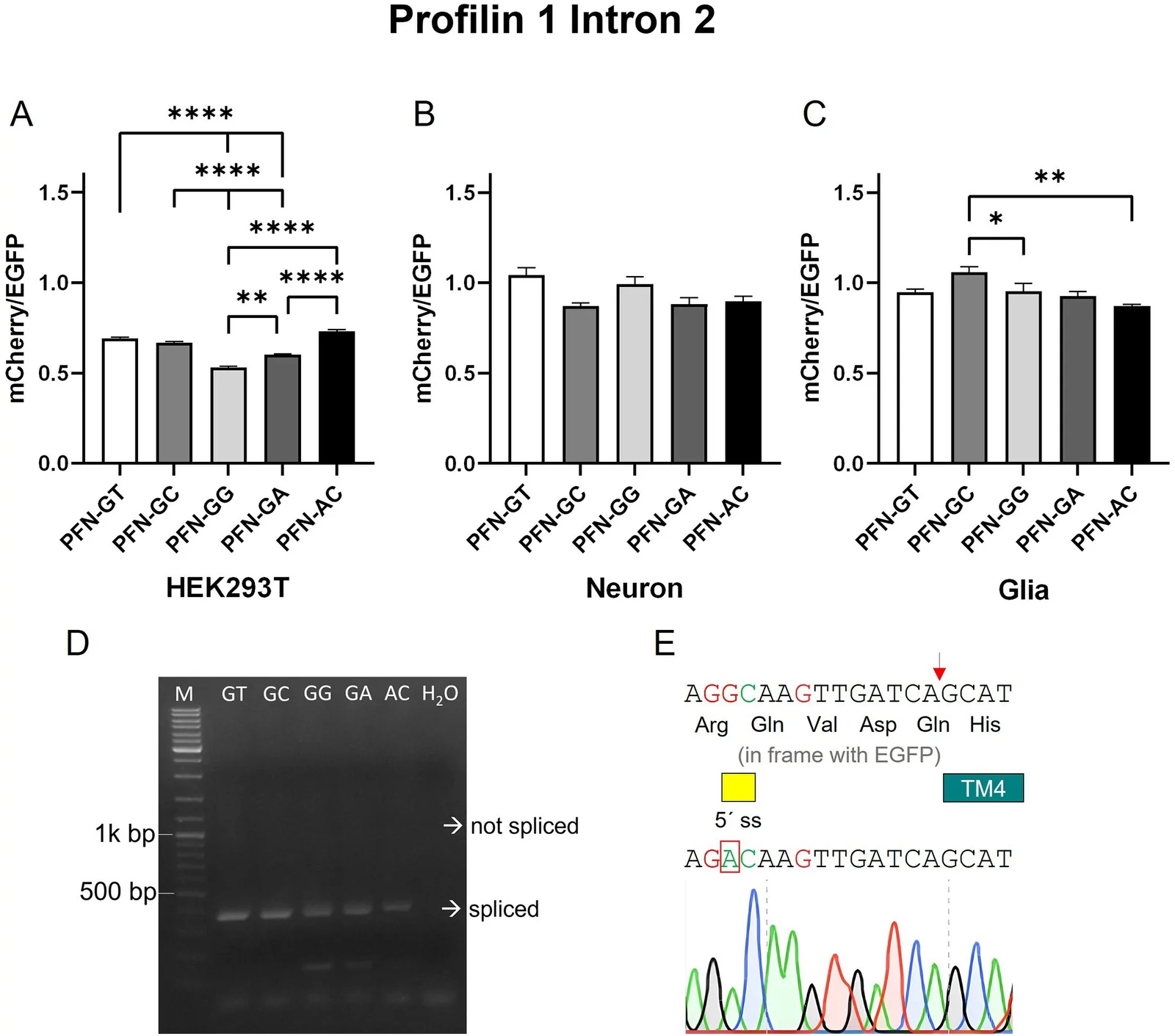
Noncanonical splice site utilization within the cryptic splice reporter with the natural PFN1 intron 2 in HEK293T cells and MHC. (**A–C**) Quantification (*N* = 3) of the mCherry to EGFP fluorescence ratio in the nucleus of transfected HEK293T cells (**A**), MHC DIV10 neurons (**B**), and MHC DIV10 glia (**C**). The ratio indicates the relative splicing of the PFN intron in the reporter constructs with GT (*n* = 730 [A]; 77 [B]; 102 [C]), GC (*n* = 393[A]; 76 [B]; 101 [C]), GG (*n* = 425 [A]; 66 [B]; 75 [C]), GA (*n* = 375 [A]; 63 [B]; 101 [C]), and AC donor (*n* = 333 [A]; 84 [B]; 87 [C]). Data are presented as mean ± SEM. *P ≤.05; **P ≤.01; ^****^P ≤.0001. (**D**) Gel electrophoresis image of PCR products obtained from cDNA of CRYSPER-transfected HEK293T cells with the PFN1 intron 2. All constructs display the 397 bp long fragment generated by splicing. The DNA ladder marker (M) is displayed on the left and adjacent are the different CRYSPER constructs, that differ by only one base pair in the splice donor as referenced above. (**E**) Sequencing results of the spliced CRYSPER-PFN-AC construct isolated from the gel above revealed the utilized 5´-splice site as a canonical donor (GU) 12 bp downstream (spliced at red arrow).

To verify that the cryptic splicing was not reliant on the synthetic intron, we exchanged it to the profilin1 intron2 (PFN). Results in HEK293T, MHC neurons and glia cells showed consistent splicing almost independent of the donor composition, where only in HEK293T cells the donor hierarchy was still noticeable (Fig. 5). Even the AC construct, initially serving as negative control, was spliced comparable to the other sensors. Sequencing of isolated cDNAs revealed the expected splicing pattern in the CRYSPERs, but in the AC vector an unexpected splice donor 12 bp downstream was activated and triggered mCherry expression (UGAUCA**GU**AUGAGUU, Fig. 5E). Thus, compared to the synthetic intron, *cis-*acting regulatory elements in the PFN intron must have driven splicing in all conditions, even if the predicted donor was not selectable in the AC construct. Interestingly, the natural intron had no influence on the choice of the nonpredicted splice acceptor site in GlyR α3 TM4.

**Figure 6.**
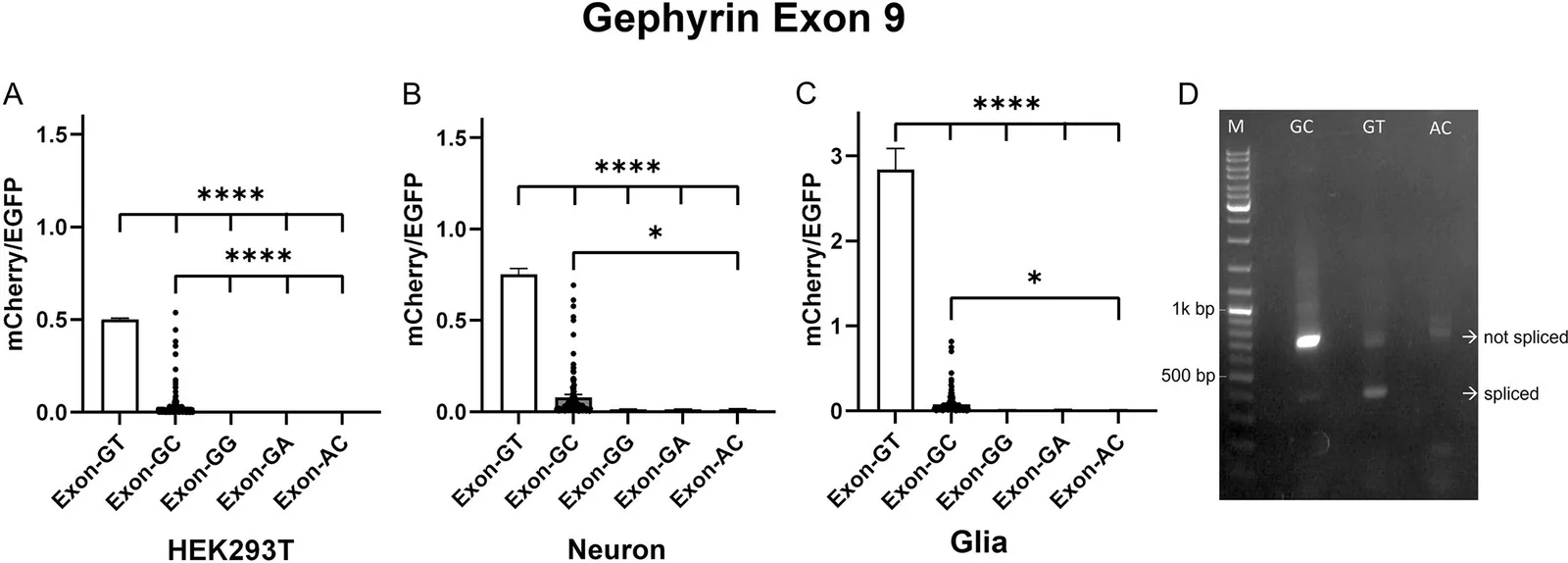
Noncanonical splice site utilization within the cryptic splice reporter with the exon 9 from *GPHN* in HEK293T cells and MHC. (**A–C**) Quantification (*N* = 3) of the mCherry to EGFP fluorescence ratio in the nucleus of transfected HEK293T cells (**A**), MHC DIV10 neurons (**B**), and MHC DIV10 glia (**C**). The ratio indicates the relative splicing of the exon 9 in the reporter constructs with GT (*n* = 397 [A]; 209 [B]; 126 [C]), GC (*n* = 313 [A]; 167 [B]; 131 [C]), GG (*n* = 189 [A]; 86 [B]; 53 [C]), GA (*n* = 212 [A]; 63 [B]; 89 [C]), and AC donor (*n* = 255 [A]; 89 [B]; 79 [C]). Data are presented as mean ± SEM. *P ≤.05; **P ≤.01; ^****^P ≤.0001. (**D**) Gel electrophoresis image of PCR products obtained from cDNA of CRYSPER-transfected HEK293T cells with the *GPHN* exon 9. Both the upper unspliced (866 bp) and lower spliced (397 bp) bands were produced. The DNA ladder marker (M) is displayed on the left and adjacent are the different CRYSPER constructs, that differ by only one base pair in the splice donor as referenced above.

To further investigate whether inherent regulatory splicing elements or the donor strength had greater influence on the cryptic splicing we exchanged the CRYSPER intron with an exon from gephyrin (*GPHN* exon 9), including an additional nucleotide at the beginning to preserve the internal STOP codon. The results demonstrate that the canonical GT splice donor was sufficient to induce splicing of the exon, while most of the noncanonical donors were not. However, the GC donor led to occasional splicing to some cell-specific extent in HEK293T cells (10.5%), MHC neurons (16.2%), as well as glia cells (14.5%) and the spliced band could be isolated from HEK293T cell cDNA (Fig. 6). Surprisingly, splicing of the GT vector was significantly increased in glia cells, with 2.84 ± 0.25 compared to 0.75 ± 0.03 in the neurons (*P* <.0001).

### Specific donor mutations abolished cryptic splice site utilization

**Figure 7.**
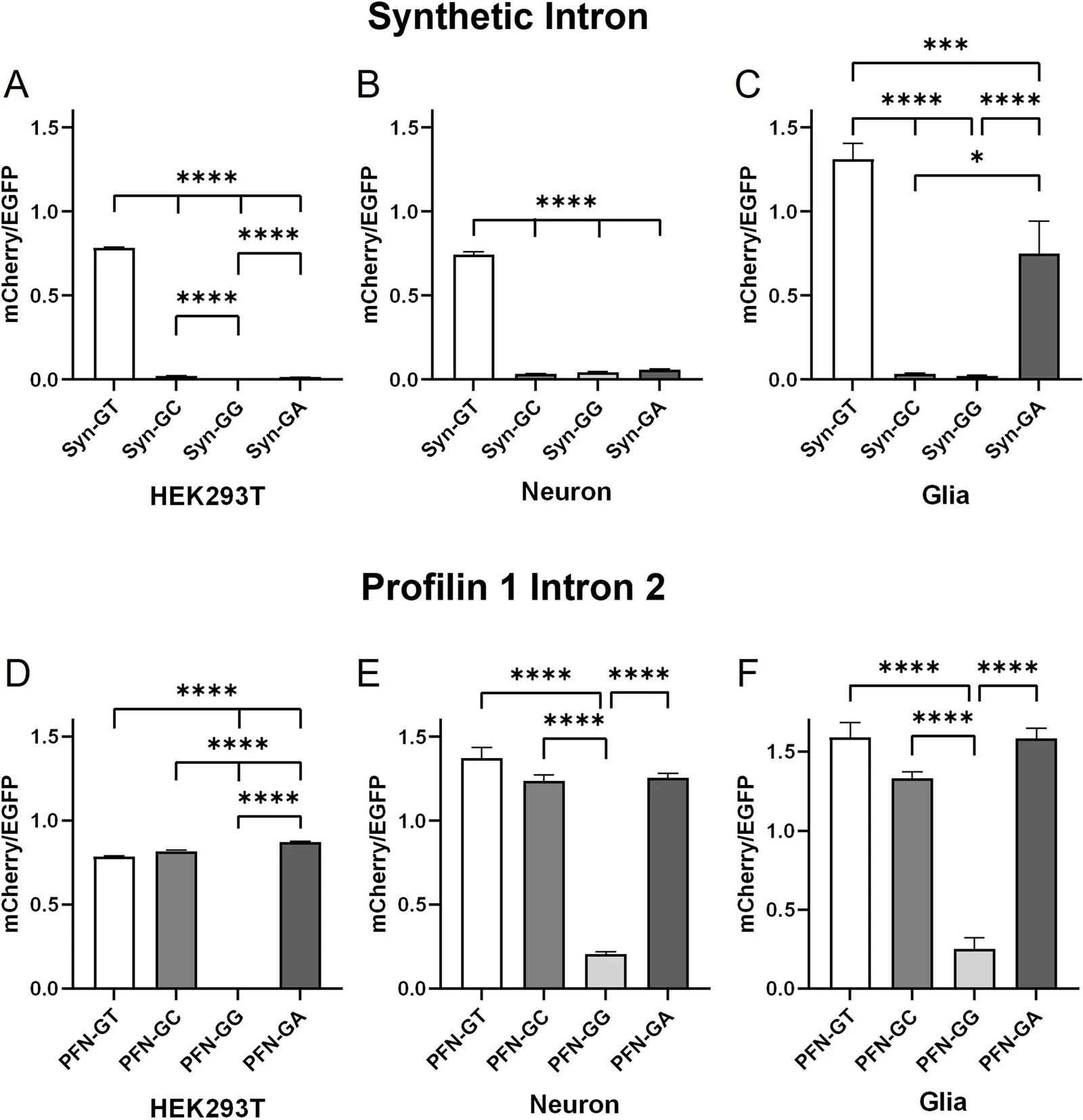
Noncanonical splice site utilization within the cryptic splice reporter with the +3/+5T mutated 5´-donor in HEK293T cells and MHC. (**A–F**) Quantification (*N* = 3) of the mCherry to EGFP fluorescence ratio in the nucleus of HEK293T cells (A/D), MHC DIV10 neurons (B/E), and MHC DIV10 glia (C/F) transfected either with the +3/+5T splice donor mutated reporter with the synthetic (A–C) or PFN intron (D–F). The ratio indicates the relative splicing in the reporter constructs with GT (*n* = 524 [A]; 120 [B]; 57 [C]; 448 [D]; 98 [E]; 88 [F]), GC (*n* = 362 [A]; 122 [B]; 48 [C]; 459 [D]; 109 [E]; 94 [F]), GG (*n* = 433 [A]; 104 [B]; 61 [C]; 382 [D]; 71 [E]; 92 [F]), and GA donor (*n* = 358 [A]; 103 [B]; 61 [C]; 399 [D]; 82 [E]; 63 [F]). Data are presented as mean ± SEM. *P ≤.05; ^****^P ≤.0001.

Next, we used splicing prediction tools (Spliceator; 40, Table 2) to predict mutations that could abolish noncanonical splicing, while maintaining canonical splicing. After introduction of +3/+5T mutations (exon–intron border as reference) in the 5´-splice donor, the mCherry fluorescence decreased significantly in cells expressing the sensors containing the synthetic intron with cryptic GC, GG, and GA donors (Fig. 7A–C). Regarding mutated AC constructs, we focused specifically on the noncanonical splice donors because the unintended alternative splice site would lead to nonspecific activation of the sensor. As anticipated, the GT donor was still utilized and interestingly glia cells still spliced with the GA donor. Note that introduction of the +3T mutation resulted in a new GT donor (GGT) in the GG construct (UUGAGG**GU**AUUUGA) which frameshifted the following ORF, thereby disrupting mCherry expression. Surprisingly, the vectors carrying the PFN intron were undisturbed by the mutations (Fig. 7D and E) against predictions (Table 2). However, cDNA isolation revealed a compensatory usage of the GT donor 12 bp downstream, as previously observed in the CRYSPER-PFN-AC construct (Fig. 5E). Moreover, an additional donor at the position V163 of EGFP (UCAAG**GU**GAACUUC) was utilized which does not enable mCherry expression. These results indicate that the presence of *cis-*regulatory elements in the profilin 1 intron are able to trigger splicing by directing the spliceosome to the nearest possible splice donor, while keeping the nonpredicted splice acceptor in GlyR α3 TM4. They also reveal that glia cells do not need *cis-*regulatory elements to enable cryptic GA splicing with the synthetic intron.

**Table 2.** Prediction of splice sites according to the Spliceator model [40]

Splice sites	Mutations	Donor composition	Model score
Splice donor 1	−1/−2/+3G	GT	0.999
		GC	0.966
		GG	0.986
		GA	0.974
	+1/+3T = (+3/+5T)	GT	0.831
		GC/GG/GA	/

### C-to-U RNA editing inducible protein expression system

From the initial results it could be suggested that C-to-U RNA editing may be reenabled by an additional mutation (+7C), which closes the *ApoB* secondary structure loop by binding to the mutated G at position −2. Even when combined with the mutated donor construct (+1/+3T) that disabled noncanonical splicing with the GC donor, no editing-dependent splicing was induced. Instead, the donor in EGFP and a novel intronic donor at position +106 (AUCUUG**GU**GAGGGG) were utilized (Supplementary Fig. S3A/B), which do not result in mCherry expression.

However, an approach with the single −1G mutation directly upstream of the editable site led to construction of a C-to-U RNA editing inducible protein expression system (CUREIPES, Fig. 1A).

**Figure 8.**
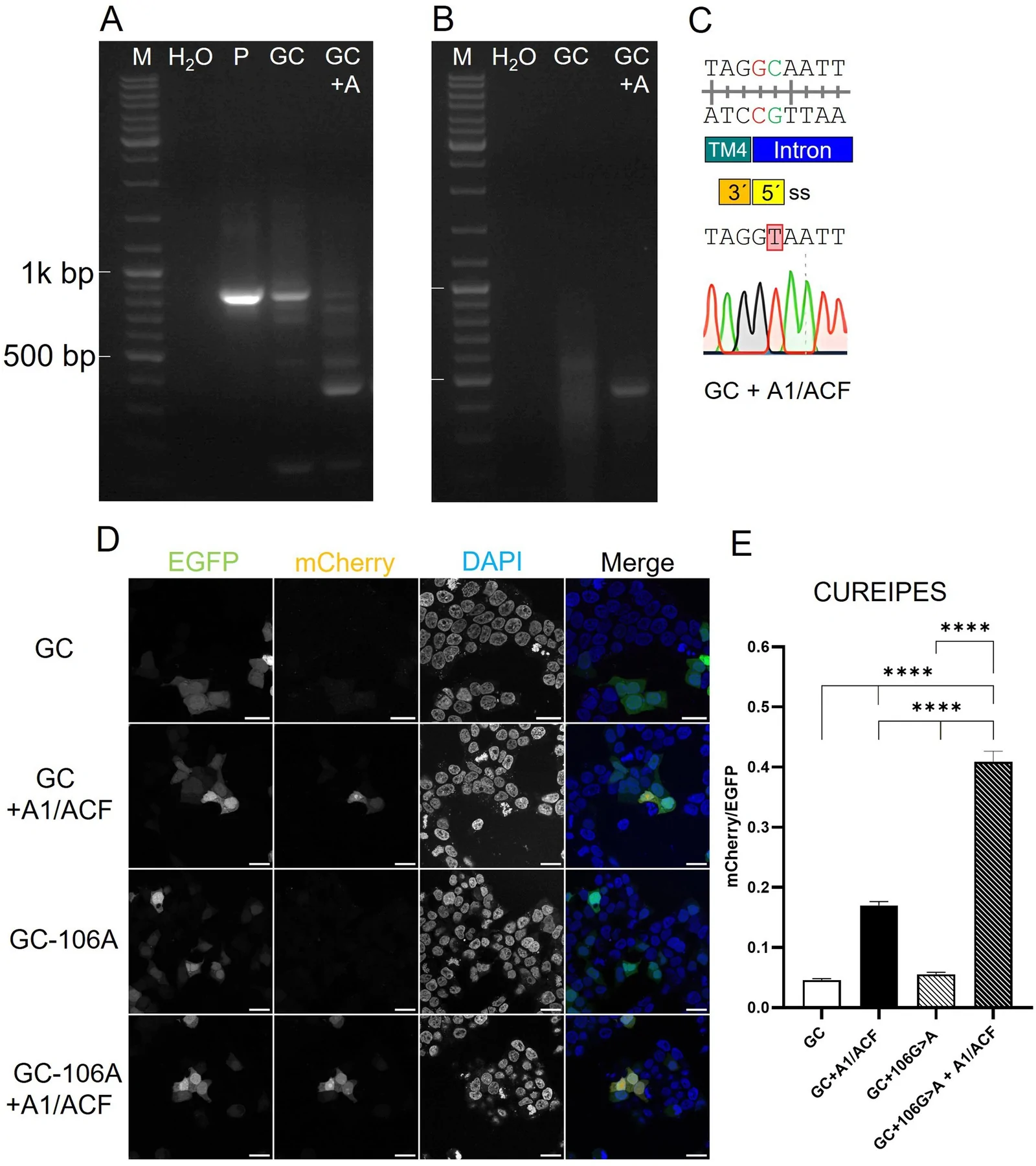
Construction of a C-to-U RNA editing inducible protein expression system (CUREIPES). (**A**) Gel electrophoresis image of PCR products obtained from cDNA of CUREIPES-transfected HEK293T cells with primers flanking the intron. The DNA ladder marker (M) is displayed on the left and adjacent are the plasmid control as well as the CUREIPES-GC constructs without and with hApobec1 and ACF overexpression. Note, that the spliced band at 397 bp is only visible when the deaminase was present. (**B**) Gel electrophoresis image after intron isolation from CUREIPES-expressing HEK293T cells revealed the 469 bp long band only in the GC setting with additional A1/ACF overexpression. (**C**) Sequencing result of this isolated intron (**B**) displaying the C-to-U RNA editing event that led to reporter splicing. (**D**) Representative fluorescence images of CUREIPES-expressing HEK293T cells with and without the intronic mutation G106A as well as additional A1/ACF overexpression. The scale bar represents 25 µm. (**E**) Quantification of the mCherry to EGFP fluorescence ratio in the nucleus of transfected cells, indicative for the relative editing induced splicing activity in the CUREIPES constructs with GC donor (*n* = 413), additional A1/ACF overexpression (*n* = 475) and the optimized construct with the G106A mutated intron (*n* = 150) and A1/ACF overexpression (*n* = 150). Data are presented as mean ± SEM. ^****^P ≤.0001.

The functionality of this vector (GC) was verified by its expression in HEK293T cells with or without hApobec1/ACF overexpression. Fluorescence analysis revealed significantly higher mCherry to EGFP ratio if the deaminase was present (Fig. 8D/E). Nevertheless, it was not to the same extend as the CRYSPER with 0.18 ± 0.01 compared to 0.65 in the GC and 0.69 in the GT vector (*P* <.0001). In addition, gel electrophoresis after reverse transcriptase-polymerase chain reaction (RT-PCR) and sequencing of the isolated bands demonstrated the spliced band only when editing was possible (Fig. 8A). Interestingly, the previously described splice patterns were also observed, together with the novel donor within the intron (Supplementary Fig. S3B), none of which induced mCherry expression. Intron isolation enabled the sequencing of the intron border and showed exclusively GT at the donor implying splicing after C-to-U RNA editing occurred (Fig. 8B/C).

To optimize CUREIPES, the novel intronic splice donor was mutated (+106 G→A), resulting in a significant increase of its efficacy to 0.41 ± 0.03 (previously 0.18 ± 0.01, *P* <.0001, Fig. 8D/E). Furthermore, all alternative splice patterns were abolished, leaving only the predicted spliced product (397 bp) upon Apobec1/ACF co-expression (Supplementary Fig. S4).

## Discussion

Here, we present new molecular tools for analysis of splicing and RNA editing, in particular noncanonical splice donor utilization. Modifications of the EdSensor [7] enabled splicing but abolished editing in the plasmid-encoded sensor, creating a cryptic splice reporter (CRYSPER). The construct can be used to investigate (noncanonical) splicing in single cells with fluorescence readout (Fig. 1).

Analysis of the influence of consensus sequence mutations on the editing efficiency revealed that mutation −1G (relative to the edited C) had no, the additional +3G a partial and −2G a detrimental effect (Fig. 2). Position −1A is conserved in mammalian *ApoB* mRNA and only guinea pig *ApoB* contains −1G and is poorly edited in rabbit liver extract *in vitro*. Although this was initially attributed to variations in the 3´-efficacy elements with a different stem-loop model and a compensatory higher editing activity of guinea pig extract [41], Maris *et al*. [11] hypothesized that it adversely affects editing by preventing access via base pairing or by creating undesirable interactions with proteins during the reaction. However, our results indicate that −1G had no significant effect on the RNA editing of the EdSensor by Apobec1/ACF in HEK293T cells, as both the ratio of nuclear to cytoplasmic EGFP fluorescence (Fig. 2A/B) and sequencing results of the edited construct were comparable to those of the control (Fig. 2C/D). The reduction of editing upon −1/+3G mutation may originate from an interaction with Apobec1/ACF or from alterations of the secondary structure, which could impair interactions or access. Switching position +3 from a pyrimidine to a purine nucleotide might have also contributed by impacting the chemical environment and potential bindings. Nevertheless, position −2 was determined to have a significant impact on RNA editing efficiency, as evidenced by exclusive cytoplasmic localization of EGFP (Fig. 2A/B) and the absence of the edited nucleotide in the sequencing results (Fig. 2C/D). Structurally, it is the initial nucleotide that pairs with the opposing mooring sequence at position +7A, thereby closing the octa-loop [11]. The −2G mutation disrupts this pairing, leading to an opened and enlarged loop. Spacer length variations have previously been shown to decrease the RNA editing efficiency [42]. Additionally, both Apobec1 and ACF bind AU-rich RNAs [11, 43] and all three mutations changed nucleotides from A or U to G, potentially affecting binding affinity. While position −2 in this splicing-dependent construct is also the last exonic nucleotide which interacts with U1 and U5 snRNP during the splicing reaction, it can be concluded that splicing and editing were mutually exclusive in this reporter.

As splicing still occurred and the noncanonical GC donor was utilized the outcome of the EdSensor modifications was a noncanonical/cryptic splice reporter (CRYSPER, Figs 1 and 3). The initial finding could be explained by extensive base pairing of the donor with U1 snRNA because mutations followed predictions and by compensation of the GC mismatch via shifting or bulging out of nucleotides [16, 23, 44]. These introduced mutations targeted residues essential for splicing, as position −1G is invariant, while positions −2 and +5 (previously +3; numbering now refers to the exon/intron border) are required for exon–intron linkage and mediate interactions with U1 (both), U5 (−2), and U4/U6 (+5) snRNAs [16, 45, 46]. Base pairing of the highly conserved intronic +5 position with U6 snRNA could have been particularly important for splice site selection. Accordingly, mutations at this position frequently activate cryptic splice sites, whereas introduction of a G at the corresponding +5 position can promote the formation of de novo splice sites [22, 46]. Moreover, the unexpected result aligns with data showing that the GC donor occurs in 0.5% of human splice sites [15]. The next experiments with the typical GT and the other cryptic GG and GA donors demonstrated noncanonical splice site usage in human and primary mouse cell cultures (Fig. 4 and Supplementary Fig. S2). As mCherry expression proves a coherent open reading frame, the splicing mechanism was not stalled as anticipated from previous studies showing that GG and GA donors impede the second spliceosomal catalyzation step [20, 21].

The observed noncanonical splicing might be attributed to unusually high donor strength but it highlights the potential presence of a tolerated level of cryptic splicing, which in case of the sensor transcript, evades nonsense-mediated decay due to the formation of a meaningful open reading frame. However, the rare occurrence of functional noncanonical donors in the human genome [15] indicates that the splice donor composition is constrained. Consequently, such deviations could serve as mechanism to achieve context-specific gene expression. The increased prevalence of these noncanonical splice sites in some organisms [19] suggests the development of a specific adaptation, accompanied by an inhibitory mechanism in others, underscoring a selective evolutionary pressure to maintain splice site fidelity. In conclusion, our findings show that noncanonical splice donors could be operational in cells with a frequency and in ways not previously realized.

The donor strength depends on an U1 snRNA:5´splice site complementary optimum, with stronger complementation decreasing splicing efficiency and with possible compensation of mis-pairs [45, 47, 48]. As the specific GC donor exhibited a similar strength to the GT donor, contiguous base pairing might play a less significant role. The mismatches of the second intronic position were compensated for but the GA and GG mutants may increase unfavorable interactions or reduce flexibility of the assembling spliceosome because of the larger purine structure. The observation that purines have a higher impact on bulges in RNA duplexes compared to pyrimidines has been reported before [49, 50]. The presence of double guanines might further amplify these effects, thereby explaining the proposed and here supported T > C > A > G hierarchy in donor preference [22]. Therefore, the second intronic position acts as a wobble base, as splicing tolerates other nucleotides to a certain extent.

This notion was also supported by the further CRYSPER application with a natural intron (PFN, Fig. 5) and together with the exon experiments (Fig. 6) demonstrated the influence of intronic splicing regulatory elements. PFN intron-containing reporters were extensively spliced across all donors and all cell types, indicative of a more efficiently spliced or regulated intron. Even in the negative control AC sensor splicing was induced with the downstream canonical donor. Splicing appeared largely independent of the donor, with noncanonical donor utilization being uniform, particularly in MHC cells. This effect could be produced by intronic regulatory elements, such as intronic splicing enhancer or secondary structures that promote splicing by recruiting *trans*-acting factors [51, 52]. These factors could have enabled the potential use of noncanonical donors, thus influencing or complementing the splice site recognition.

To avoid potential effects from intronic splicing regulatory elements, the sensor was also modified to include an exon instead of an intron. This reduced reporter splicing across all donor conditions, suggesting the presence of intronic splice enhancers in the synthetic and PFN introns or factors that enforce exon definition. Although canonical splicing with the GT donor declined, it remained detectable, reflecting the donor´s inherent strength and effective recognition by the spliceosome. In contrast, noncanonical splicing was completely abolished, demonstrating that cryptic splice site usage may depend largely on regulatory elements and their binding proteins rather than on splice site strength alone. Nevertheless, a small subset of cells still utilized the GC donor to a limited extent. This uneven splicing distribution across cells might point to regulated control rather than uniform error, which would occur more regularly. Interestingly, glia cells exhibited increased splicing of the exon which may indicate cell-type specific splicing regulation, perhaps indicating the presence or absence of a unique splicing factor or differences in error control. Therefore, the exon construct could be utilized to evaluate the inherent donor strength. Meanwhile, the same unpredicted splice acceptor in GlyR α3 TM4 was used invariantly across all constructs and cell types, indicating lack of flexibility and contrast with splice site predictions. Thus, also the exceptionally strong GlyR α3 TM4 splice acceptor could be used to oppose exon skipping. Moreover, the GlyR α3 TM4 splice acceptor could naturally be used in cells because it is located ∼210 bp downstream of the splice acceptor in the intron that separates exons 8 and 9 (NCBI, NM_006 529), eventually leading to truncated, nonfunctional, GlyR α3 protein variants.

Mutations at splice sites affect their recognition and influence the selection of cryptic splice sites [22]. Our next approach investigated the impact of mutations on donor strength and noncanonical splicing as well as resulting compensatory strategies (Fig. 7). In silico predictions (Table 2; 40) revealed +3/+5T splice donor mutations to abolish cryptic splicing while preserving canonical splicing. Notably, the +3T mutation created a new frameshifted GT donor in the GG construct, preventing mCherry expression. Despite predictions that the mutated GT splice donor would be weaker than the original, it underwent more frequent splicing with the synthetic intron in HEK293T cells, maintained the same level in glia cells and showed reduced activity only in neurons. Additionally, a subset of glia cells was identified to splice the synthetic intron with the GA donor. These results suggest a significant cell-type specific influence or context-specific cryptic splicing that current prediction models do not account for. Furthermore, the minimal differences in prediction values do not consistently translate to *in vivo* conditions and should be interpreted carefully.

Generally, the +3/+5T mutation disrupted noncanonical splicing of the synthetic intron. This aligns with a study indicating that the +5 position is crucial for selecting authentic splice donors. Following mutations at this site, splicing at adjacent cryptic splice sites became particularly common, possibly due to sequential interactions with U1 and U6 snRNAs [22]. Although approaching from a different perspective, the selection of a splice donor and its competition with nearby cryptic splice sites appears to depend on this specific site. Additionally, position +3 was altered to a nucleotide that cannot base pair with pseudo uridine, thereby enlarging the mismatch to U1 snRNA following only the noncanonical dinucleotides. The presence of a two-nucleotide bulge increases detriment due to the loss of base stacking and the introduction of a kink, which enlarges the helix angle [44, 50, 53].

Alteration of the splice donor led to compensatory splicing patterns which might showcase the interplay between splicing regulatory elements and splice sites. While the mutation abolished the original splice donor, regulatory factors still induced splicing, utilizing adjacent splice sites. For instance, the PFN intron was spliced by activating the weaker donor located 12 bp downstream and for the synthetic intron a donor inside the EGFP-coding region was utilized. This would also be consistent with the role of U6-snRNA, as reverting +5G led to splicing at the nearby alternative donor sites located upstream within EGFP (Syn intron) and downstream at position +12 (PFN intron). In conclusion, the prediction values are imprecise and sometimes fail to indicate the correct outcome due to the absence of context-specific parameters, such as cell type and regulatory factors. However, the sensor presented here could potentially address this limitation by providing a relative splicing amount, and by combining this readout with measurements across different cell types or under varying regulatory factor conditions, allow the definition of more biologically grounded input parameters to improve the splice site predictions.

Established minigene splicing reporter assays typically involve transfecting vectors that contain two or more exons separated by introns, followed by RT-PCR and sequencing to analyze splicing events [54]. These assays can also employ single or dual fluorescent or bioluminescent proteins, spliceosome-mediated RNA *trans*-splicing, ribozyme, or radionucleotides as means of reporting information. Such outputs aid in screening for splicing inhibitors and regulators and in studying splice sites, splicing efficiency and introns [55]. The fluorescence-based CRYSPER described here offers the advantage of single cell analysis compatible with high throughput flow cytometry (Supplementary Fig. S5) and without the need for RNA extraction, making it fast, easy, and cheap to use. It includes a normalization marker that allows it to maintain unaffected by variations in transcription, transport, mRNA stability, translation, and transfection efficiency. It also establishes a basis for single cell RNA sequencing to advance our understanding of cell type-specific regulations of RNA splicing. As each intron, sequence combination and cell type exhibit distinct behavior, it is difficult to derive general rules. Although detailed investigation of individual cases uncovered mechanistic insights specific to their cellular contexts, it does not allow conclusions about whether specific nucleotides generally fulfill analogous functions across different systems. Nevertheless, CRYSPER provides a valuable starting point for the development of splice donor-, intron-, and cell type-tailored systems to address specific questions across different sequence- and cell type-specific contexts.

For the initial purpose of our study, that was a C-to-U RNA editing inducible protein expression system (CUREIPES, Fig. 1), a single point mutation of the Apobec1 consensus sequence (−1A→G) was sufficient (Fig. 8). In fact, sequencing the intron revealed exclusively the edited nucleotide, with no evidence for noncanonical GC splicing. Additional −2G mutation abolished editing, but was insufficient to create a donor strong enough to permit noncanonical GC splicing (Fig. 2). Introduction of the subsequent +3G mutation produced a sufficiently strong donor, rendering splicing independent of editing (Figs 1C and 2). The +7C mutation did not restore editing (Supplementary Fig. S3A/B), likely due to altered protein interactions or because the stem loop melting requires an A-U pair at the beginning of the loop. Thus, in CUREIPES, removal of the intronic stop codon is dependent on and occurs only after hApobec1/ACF-dependent RNA editing has generated the canonical splice donor site. Initially, expression efficacy remained relatively low due to the utilization of an alternative splice site, but mutation +106A eliminated the intronic donor and significantly increased editing-dependent protein expression (Fig. 8C/D and Supplementary Fig. S4).

CUREIPES offers the advantage of expression initiation rather than terminating translation after C-to-U editing by hApobec1. As mCherry can be replaced by any protein-coding sequence of choice, it can be used to restore homeostasis when editing levels are elevated via the expression of proteins known to inhibit editing, such as SYNCRIP (GRY-RBP) [43], hnRNP-C [56], or CELF2 (CUGBP2) [57]. Likewise, regarding reduced editing levels for example in atherosclerosis, CUREIPES-dependent Apobec1/ACF expression could counteract. Moreover, it could be utilized to investigate the interactions within the editing network by enabling precise protein expression. Here, C-to-U RNA editing depends on ACF and could be adapted for other cofactors, such as RBM46 [58] or RBM47 [59], to enable intervention and analysis of their sequence requirements. Other applications include for example C-to-U RNA editing-dependent conditional protein in animal models if mCherry is replaced by the Cre-recombinase.

Taken together, CRYSPER and CUREIPES are versatile tools that will contribute to our understanding of RNA processing in health and disease.

## Supplementary Material

gkag899_Supplemental_Files

## Data Availability

All experimental data are available as supplementary Excel files.
