# Supplementary material for: CRYSPER and CUREIPES: two new molecular tools enabling analysis of RNA splicing and C-to-U RNA editing-inducible protein expression at single cell resolution
Source: Nucleic Acids Res. 2026 Sep 24;54(18):gkag899. doi: 10.1093/nar/gkag899 (PMC13601067; doi:10.1093/nar/gkag899)
Supplement: Supplementary file 1 [file gkag899_supplemental_files.zip › Harnau et al_Supplementary Information_MH_JCM.docx]

**Supplementary Information**

**CRYSPER and CUREIPES: Two new molecular tools enabling analysis of RNA splicing and C-to-U RNA editing-inducible protein expression at single cell resolution**

Magnus Harnau^1^, Yulia Novykova^1^*, Jacqueline Weicht^1^*, Barbara Schweissthal^1^, Leonie Emde^1^, Julia Brach^1^, Josephine Haake^2^, Laura Steenpass^2^, Steffen Fricke^1^, Jochen C. Meier^1,3^

1) Division Cell Physiology, Institute for Cell- and Neurobiology, Technical University Braunschweig, Spielmannstr. 7, D-38106 Braunschweig, Germany

2) Department Human and Animal Cell Lines, Leibniz Institute DSMZ - German Collection of Microorganisms and Cell Cultures GmbH, Inhoffenstr. 7b, 38124 Braunschweig, Germany

3) Correspondence: [jochen.meier@tu-braunschweig.de](mailto:jochen.meier@tu-braunschweig.de)

*) Equal contribution

**Supplementary Figures:**

5’-***GC****AA****G****TTGATCAGTATt*ctctagcctatccatca**tag**ccagagaaggcccgaatgccctttctgccgttggcccttTTTTTCACCTCTACAAACATTTAGCATCTTGGTGAGGGGTGGGGAAGAGTTGCTTTTTCTTTATGCCATTGATATTTGGCTATGTTAGAATATTATTTAATTCTCAGTTATCATAGTGAATTAAAAATTTATATGTATCAGATAATCACCATCAGATGGTACCGATCAAGAGTGAAACTGTTCCATGAATTGTGCTTAGAAATTTTCTTACTGACTGTTGAAAAGAATCTGTGTTTCCTCGTTGAATACAAAGTGTCCTAGGACTGGGATGTTTTGAGCAAGCAGGCAAATTTTTCTGCACATGACTATACTATTGTTTTCTTTCAG*GGCCTGCTTTCCTCTAGCATTTCTGATTTTTAACATTTTCTACTGGGTTATCTATAAAATCCTTAG*-3’

**Figure S1: Synthetic intron sequence**. At 5‘, the yellow highlighted sequence in italic capital letters correspond to the -1/+3G (red) mutated Apobec1 consensus sequence. The C in green is position 0 which is C-to-U RNA-editable. Small letters correspond to the sequence of the *Glrb* intron, while capital letters downstream show the sequence derived from *GPHN* introns as described in the main text (highlighted dark blue). The STOP codon tag is shown in red. The predicted splice acceptor site AG is highlighted in green. The actually used spliced acceptor in the TM4 region in italic capital letters (blue-green) is highlighted in pink. The color code corresponds to the schemes shown in graphical abstract and Figure 1.

**Figure S2: Representative images** **of the cryptic splice reporter in MHC DIV10 neurons and glia cells for the fluorescence readout analysis.** **(A/B)** Representative images of MHC DIV10 neurons (A, MAP2^+^) and glia cells (B, MAP2^-^) expressing the cryptic splice reporter with the synthetic intron and the different splice donor sites. The scale bars represent 50 µm.

**Figure S3: Attempts to restore the octa-loop did not result in a functional C-to-U RNA editing inducible protein expression system (CUREIPES). (A)** Gel electrophoresis image of PCR products obtained from cDNA of CUREIPES +1T/+3T/+7C-transfected HEK293T cells with primers flanking the intron. The DNA ladder marker (M) is displayed on the left and adjacent are the GC constructs without and with hApobec1 and ACF overexpression as well as the plasmid and water control. Note that the predicted spliced band at 397 bp is absent, while the spliced band generated with the donor in EGFP (158 bp) is prominent and a new band at 504 bp appeared. **(B)** Sequencing of this new spliced band (A, 504 bp) revealed no C-to-U RNA editing and a newly emerged splice donor downstream of the intron (GT at intronic position +106). However, this splicing pattern would not result in protein expression, as it is located downstream of the internal stop codon.

**Figure S4: Optimization of the C-to-U RNA editing inducible protein expression system (CUREIPES).** Gel electrophoresis image of PCR products obtained from cDNA of CUREIPES -1G/+106A-transfected HEK293T cells with primers flanking the intron. The DNA ladder marker (M) is displayed on the left and adjacent are the CUREIPES-GC+106A constructs without and with hApobec1 and ACF overexpression as well as the plasmid and water control. Note, that the predicted spliced band at 397 bp is only visible when the deaminase was present.

**Figure S5: Verification of the CRYSPER results with flow cytometry of transfected HEK293T cells. (A/B)** Representative measurements of HEK293T cells expressing the various CRYSPER (A) and +3/+5T donor mutant CRYSPER (B) constructs. The graphs depict the mCherry fluorescence of EGFP positive cells. The mCherry negative cells are marked in green and the positive cells in red. **(C)** Quantification of the percentage of the EGFP positive cell population that were also mCherry positive. Data are presented as mean ± SEM.
